# Empowering prediction of miRNA–mRNA interactions in species with limited training data through transfer learning

**DOI:** 10.1016/j.heliyon.2024.e28000

**Published:** 2024-03-15

**Authors:** Eyal Hadad, Lior Rokach, Isana Veksler-Lublinsky

**Affiliations:** Department of Software and Information Systems Engineering, Ben-Gurion University of the Negev, David Ben-Gurion Blvd. 1, Beer-Sheva 8410501, Israel

## Abstract

MicroRNAs (miRNAs) play a crucial role in mRNA regulation. Identifying functionally important mRNA targets of a specific miRNA is essential for uncovering its biological function and assisting miRNA–based drug development. Datasets of high-throughput direct bona fide miRNA–target interactions (MTIs) exist only for a few model organisms, prompting the need for computational prediction. However, the scarcity of data poses a challenge in training accurate machine learning models for MTI prediction. In this study, we explored the potential of transfer learning technique (with ANN and XGB) to address the limited data challenge by leveraging the similarities in interaction rules between species. Furthermore, we introduced a novel approach called TransferSHAP for estimating the feature importance of transfer learning in tabular dataset tasks. We demonstrated that transfer learning improves MTI prediction accuracy for species with limited datasets and identified the specific interaction features the models employed to transfer information across different species.

## 1 Introduction

MicroRNAs (miRNAs) are short non-coding RNAs (~22 nts) that regulate gene expression post-transcriptionally. They play critical roles in developmental, physiological, and pathological processes [1], [2]. miRNAs are produced from primary miRNA transcripts (pri-miRNAs) by a multistep biogenesis pathway [3]. The mature, functional miRNAs associate with Argonaute proteins to form a miRNA-induced silencing complex (miRISC). This complex interacts with miRNA complementary binding sites on 3' UTRs of target mRNA, which leads to translation repression and/or degradation of the target mRNA [4]. Identifying functionally important mRNA targets of a specific miRNA is essential for uncovering its biological function and assisting miRNA-based drug development. Thus, high-throughput experimental methods (e.g., [5], [6], [7], [8]), as well as advanced machine learning (ML)-based computational methods (e.g., [9], [10], [11]), have been developed in recent years for this task (for a review see [12]).

Experimental protocols like cross-linking, ligation, and sequencing of hybrids (CLASH) [7], covalent ligation of endogenous Argonaute-bound RNAs (CLEAR)-CLIP [8], [13], and modified iPAR-CLIP [14], which include a ligation step, can produce high-throughput, unambiguous interacting miRNA–target datasets. However, due to technical challenges involved in applying these methods, datasets of direct bona fide miRNA–target interactions (MTIs) exist only for a few model species. Thus ML-based tools are limited by the datasets that can be used for training.

Previously published ML methods showed the possibility of predicting MTIs in one species using a model trained on data from a different species [15], [16], [17]. However, in these methods, training was performed on human datasets only. In our recent research [12], we performed intra- and cross-species classifications using datasets from human, mouse, worm, and cattle, considering all possible combinations, where each dataset served as the source (training) and the target (testing). As expected, the performance of cross-species classification is typically lower than intra-species classification. We found that the transferability of miRNA targeting rules between different species depends on several factors, including their evolutionary distance. Furthermore, recent research [18] addressed the MTI prediction by incorporating features related to protein products of the target genes. Similarly, their cross-species experiments revealed shared principles in miRNA regulation across species and this generalization drops as the evolutionary distance between species increases. However, it has not yet been explored whether cross-species classification can be enhanced by enriching the training dataset with interactions from the target species via the transfer learning technique. This direction is important since many species lack sufficient data to train an ML model from scratch.

Training set distribution is a crucial factor in ML performance. If the training set is not in the same distribution as the testing set or the real-world data, the ML model may not be able to generalize well and may perform poorly [19]. It is important to note that mixing observations from a different distribution with the training set and retraining the model can significantly damage the performance of the ML model [20]. This is because the model will not be able to learn the appropriate patterns and relationships between features and labels. To overcome those distribution differences, techniques such as transfer learning have been developed. The transfer learning method refines models already trained with data that may have come from different distributions. It focuses on transferring the knowledge gained while solving one problem to a different but related problem [21], [22]. This allows researchers to leverage knowledge acquired from one task to enhance the performance of models trained on limited data of a similar task. The significance of transfer learning extends beyond traditional domains and has found compelling applications in the burgeoning field of machine learning, particularly within the realm of biology. For instance, in the domain of tumor cancer classification, transfer learning has been harnessed to enhance the accuracy of predictions by incorporating tumor observations sourced from different data distributions [23].

In this study, we used miRNA–target interaction datasets obtained from previous research [12] to determine how to transfer interaction rules learned from a large dataset of one species to small datasets of other species. We used transfer learning methods to examine the performance of a model that was pre-trained on a large dataset and then refined on a dataset with limited interactions. We compared two machine learning methods, namely Artificial Neural Network (ANN) and Extreme Gradient Boosting (XGB), using a variety of classification evaluation metrics, as detailed below. In addition, we introduce a novel approach called TransferSHAP for estimating the feature importance of transfer learning in tabular dataset tasks. Utilizing this method in our study, we identified the specific interaction features the models employed to transfer information across different species.

Overall, our study provides significant contributions in two areas related to MTI prediction. Firstly, we propose a methodology to enrich the MTI task, allowing its application to species with few interaction observations. Secondly, we introduce a novel approach for estimating the feature importance of transfer learning. These contributions are discussed in detail in the following sections.

## 2 Materials and methods

### 2.1 Datasets

We used eight different datasets from four species: human, worm (*C. Elegans*), mouse, and cattle (*B. Taurus*), obtained from our previous study [12]. Briefly, these datasets were processed and transformed into a standard format that includes metadata (interaction ID, interaction source), information about the miRNA (e.g., name and sequence), and the target mRNA (e.g., target site sequence). RNAduplex [24] was applied to calculate interaction duplexes between miRNAs and target site sequences. Then, each interaction was classified based on the seed type: canonical seed, non-canonical seed, and “other”. Canonical seed interactions are interactions with exact Watson–Crick pairing in positions 2–7 or 3–8 of the miRNA. In contrast, non-canonical seed interactions may contain GU base pairs and up to one bulged or mismatched nucleotide at these positions. Only canonical and non-canonical seed interactions that fell within 3'UTRs of mRNA sequences, designated as positive interactions, were included in the final datasets (Table 1).

**Table 1:** Summary of eight datasets, across four species, used in this study. The datasets are designated as in [12]. For each dataset, we provide the cell type/developmental stage, the experimental method used to obtain the data, and the size corresponding to the number of positive/negative interactions.

Species	Dataset	Cell type ﹨dev. stage	Experimental method	Ref.	Dataset size	Total size per species
**Cattle**	ca1	MDBK	CLEAR-CLIP	[13]	18204	18204

	h1	HEK293	CLASH	[7]	5137	
	h2	Mix 6 datasets	AGO-CLIP endogenous ligation	[14]	1150	
**Human**	h3	Huh-7.5	CLEAR-CLIP	[8]	2846	9133

	m1	Mix 3 datasets	AGO-CLIP endogenous ligation	[14]	537	
**Mouse**	m2	N2A (ATCC)	CLEAR-CLIP	[8]	17574	18111

	ce1	L3	Modified iPAR-CLIP	[14]	1176	
**Worm**	ce2	Mid-L4	ALG-1 iCLIP endogenous ligation	[26]	992	2168

Every positive miRNA–mRNA interaction was complemented with synthetically generated negative interaction, by shuffling the mature miRNA sequence until the sequence at positions 2–7 and 3–8 in the shuffled miRNA did not match with the same regions of any real miRNA of the examined species (according to miRBase [25]) and identifying the most favorable target sequence within the entire 3'UTR sequence by RNAduplex [24]. This procedure resulted in balanced datasets with the same ratio of positive and negative interactions. All positive and negative interactions are characterized by 490 numeric and Boolean features extracted from each miRNA–mRNA pair. All the above processing procedures and features are explained in detail in [12]. To perform the experiments on the “species” level and increase the size of the datasets, we merged the eight datasets into four unified datasets as indicated in the last column in Table 1.

#### 2.1.1 Splitting the data into training and testing sets

The approach for dataset split into train-test sets has a crucial role in ML model evaluations. In this study, we partitioned each dataset into training and testing sets using an 80%-20% ratio, with 10% of the training set reserved for validation. Consequently, the validation set size remained fixed at 8% across all experiments. To maintain consistency, we employed a stratified random split algorithm, ensuring that the miRNA distribution in the training, validation, and testing sets mirrored that of the entire dataset. miRNAs with a single interaction in the data were included in the test to force the model to predict interactions of unseen miRNA sequences. This procedure was repeated ten times, yielding ten training sets and their corresponding ten testing sets for each dataset. The results are reported as average scores across all ten experiments.

**Figure 1:**
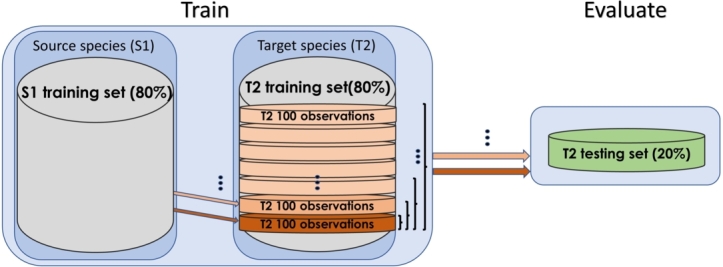
Dataset split illustration in a transfer learning experiment. For every two species, source (S1) and target (T2), an ML model was initially trained using 80% of the S1 dataset. In the transfer phase, chunks of 100 interactions (positive and negative) were used to retrain the model. 20% of the target species was used for evaluation.

In this study, we performed three types of experiments: intra-species, cross-species, and cross-species with transfer. In the *intra-species* experiments, each dataset is trained-tested with the 80-20% split. All *cross-species* experiments involve two species, a source species and a target species. Thus, we used 80% of the source species dataset for training and 20% of the target species dataset for testing. In the *cross-species transfer learning* experiments, we used 80% of the source species dataset for training the initial model. Then, in the transfer phase, we added up to 500 interactions from the training set of the target species, 100 interactions in each iteration. For evaluation, we used the 20% unseen testing set of the target species (Fig. 1). In the latter experiments, we used one of the ten 80-20% splits of the datasets.

### 2.2 Models

**Figure 2:**
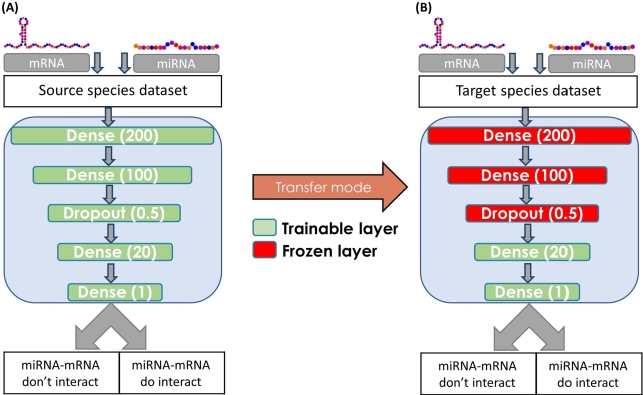
**(A) ANN Model architecture.** Every horizontal line represents a layer; dense layer parameters (in brackets) represent the number of neurons; dropout layer parameters (in brackets) represent the probability of removing a neuron during the training stage. The activation function is Relu [27] for all layers except for the last layer, in which we used Sigmoid, which outputs the probability for the interaction. **(B) ANN Transfer Learning model Architecture.** The same architecture as in (A), where some of the layers are frozen (red layers) and thus retain the parameters obtained from the previous training process.

In this study, we examined whether the transfer learning technique can enhance the cross-species classification task of miRNA–target interactions. The transfer learning method consists of two stages: (1) training the **source model** on a given source dataset and (2) conducting additional training, known as **target model** training to the source model, on the target dataset. Typically, the target dataset is smaller than the source dataset. We tested this technique using two ML models: (1) an artificial neural network (ANN) model that is commonly used in transfer learning tasks (Fig. 2A) and (2) a gradient boosting tree-based method, specifically Extreme Gradient Boosting (XGB), that was used in a previous study [12]. The implementation details, along with the code repository for all the methods described, are provided in the availability section below.

#### 2.2.1 Artificial neural network (ANN) model

We used ANN as our primary method because of its good performance on many ML tasks and its suitability for the transfer learning task. However, ANN's notable limitation is the need for an extensive training dataset; otherwise, the network tends to overfit, which leads to a decreased performance on the test set. Another ML model training challenge in general and especially in ANN is underfitting, which arises from insufficient training data. As in our study, the datasets were relatively small for ANN, we applied ML techniques related to the architecture and the parameters to minimize the overfit and the underfit.

In the ANN transfer learning technique, we stored the parameters of the network models trained on the source dataset. Then, we retrained part of the network on the target dataset, by freezing some layers such that their parameters were not updated during the back-propagation, as shown in (Fig. 2B).

#### 2.2.2 Extreme Gradient Boosting (XGB) model

We chose XGB because boosting algorithms tend to excel in small dataset tasks. They combine weak models created one after the other, and each weak model is made based on the previous models' errors. XGB needs less training data compared to neural networks. On the other hand, it sometimes fails to learn complex characteristics, while networks do it successfully. Transfer learning with XGB is done by selecting the newly created tree features concerning previously trained trees, a technique known as partial fit.

#### 2.2.3 Optimal hyperparameters search

Hyper-parameters directly control the behavior of an ML model and significantly impact the performance; therefore, choosing appropriate hyper-parameters is crucial. The hyperparameter selection process varied for each model. For the ANN, we determined the optimal architecture for both the network and the transfer learning phase through trial and error. Grid search was employed to identify the best ANN hyperparameters, such as epochs, optimizers, and activation functions. Furthermore, we explored the ideal transfer learning architecture, including the layers to freeze, as depicted in Fig. 2B.

To avoid the ANN underfitting due to the small number of observations, we performed many epochs to overcome this difficulty and enable the network weights to reach significant values. Furthermore, to avoid overfitting, we added a dropout layer between dense layers (architecture) and limited network weight values by using regulatory functions such as *l2 norm* (parameters). By using these techniques, we were able to train an ML model with many epochs on small datasets and still avoid overfitting even in deep networks, as shown in our ANN architecture (Fig. 2A). For XGB, we used a grid search algorithm to determine the optimal hyperparameters. Following the approach in our previous research [12], we evaluated seven well-known XGB parameters, including max depth, min child weight, etc., within their standard ranges.

### 2.3 Evaluation metrics

We evaluated our models using a diverse set of classification evaluation metrics, encompassing the traditional accuracy metric (ACC), the Area Under the Curve (AUC), the F1-score [28], and the Matthews Correlation Coefficient (MCC) [29]. The choice of ACC is grounded in the dataset's balanced representation of positive and negative classes, providing a meaningful measure of overall accuracy. Additionally, we employed the AUC metric due to its widespread use in classification assessments. For a comprehensive evaluation, we utilized the F1-score, a composite metric combining precision and recall (sensitivity). This metric prioritizes precision, highlighting the importance of True Positives (correctly identified interactions). The F1-score is calculated using the formula: $F1score=\frac{\text{Precision}\times \text{Recall}}{\text{Precision}+\text{Recall}}$ or $F1score=\frac{\text{TP}}{\text{TP}+0.5(\text{FP}+\text{FN})}$.

To address the consideration of other metrics, such as specificity (true negatives and false positives), we used an additional metric, the MCC. This metric offers a more comprehensive assessment, considering true positives, true negatives, false positives, and false negatives in binary classification scenarios. The MCC is calculated using the formula: $MCC=\frac{\left(\text{TP}\times \text{TN})-(\text{FP}\times \text{FN}\right)}{\sqrt{\left(\text{TP}+\text{FP})\times (\text{TP}+\text{FN})\times (\text{TN}+\text{FP})\times (\text{TN}+\text{FN}\right)}}$.

### 2.4 TransferSHAP

Feature contributions in ML are crucial in understanding the reasoning behind predicted observations. Various tools such as Shapley Additibve exPlanations (SHAP) [30], LIME [31], and InterpretML [32] were developed to calculate a model's feature importance. However, these tools do not fully support transfer learning models as they do not consider the source model's feature importance. A feature that receives a high score in the target model but also gets a high score in the source model is not essential for transfer learning despite appearing significant in standard feature importance tools.

We developed a novel method for estimating feature importance in tabular data called **TransferSHAP**. This method considers the feature importance of both the source and target models to identify features that contribute significantly to transfer learning. The TransferSHAP method first calculates the Shapley values [33] for both the source and target models. Shapley values represent the contribution of each feature to the prediction of each observation. The resulting Shapley values matrix has observations as rows and features as columns, with each cell indicating the impact of a specific feature on the model's decision. Positive values indicate contribution to the positive class, and negative values indicate contribution to the negative class. The TransferSHAP method subtracts the Shapley values of the target and source models, calculates the absolute values for both positive and negative classes, and then sums the values of each column to obtain a list of features and their importance. TransferSHAP can effectively identify features that transfer knowledge from the source to the target model.

In our study, we utilized the TransferSHAP approach to determine the interaction rules acquired by the target model during its training. We employed the TransferSHAP method to each <source, target> species pair to generate a list of the most significant features for the target model. In our research, we used Spearman correlation [34] between the TransferSHAP output vectors to discern the similarities and dissimilarities between the acquired rules.

## 3 Results

We conducted three types of analyses to evaluate whether transfer learning provides an advantage in cases where there is not enough data to train a new ML model, denoted as **intra merged species ML**, **cross species ML**, and **cross species transfer ML**.

### 3.1 Intra-merged-species ML

Our data consists of 8 datasets across four different species. In our previous work [12], we evaluated the performance of ML-based binary classifiers to correctly classify positive and negative MTIs intra- and cross-datasets, where the training and testing sets originate from the same dataset or different datasets, respectively. In this work, we first evaluate whether merging the eight datasets into four species datasets affects the ability to predict interactions intra-species.

**Figure 3:**
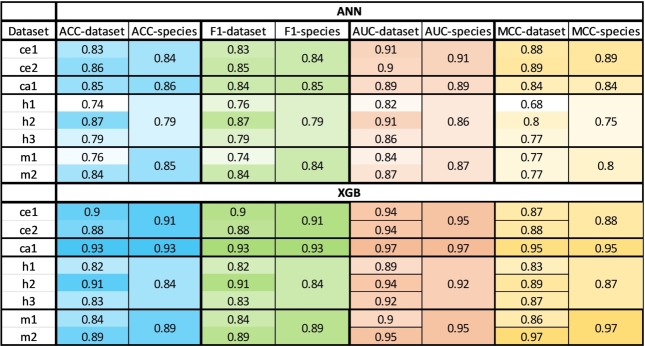
Intra-datasets and intra-merged-species classification results. The performance of both ML methods (ANN and XGB) with four evaluation metrics (ACC/F1/AUC/MCC) for the individual datasets and datasets merged by species. Each cell represents the mean metric (ACC/F1/AUC/MCC) score of the ten classifiers (ANN/XGB) that were trained and tested on different training-testing dataset splits. Heatmap color scale corresponds to the minimum and maximum values for each metric. ACC = Accuracy.

Based on the mentioned evaluation metrics, our results indicate that combining datasets typically enhanced the classification performance for species compared to datasets, with one exception for h2 dataset (Fig. 3). Taking mice as an example, the ANN achieved ACC of 0.76 and 0.84 for datasets *m1* and *m2*, respectively, compared to 0.85 for the merged mouse dataset. Similar trends are observed across the F1-score, AUC, and MCC, with values of 0.74 and 0.84 compared to 0.84, 0.84 and 0.87 compared to 0.87, and 0.77 and 0.77 compared to 0.8, respectively. Notably, the XGB model consistently outperformed the ANN model in all intra-species predictions, achieving scores of 0.89, 0.89, 0.95, and 0.97 for ACC, F1-score, AUC, and MCC on the merged mouse dataset. Therefore, in the following experiments, we used the merged species datasets.

### 3.2 Cross-species-ML

**Figure 4:**
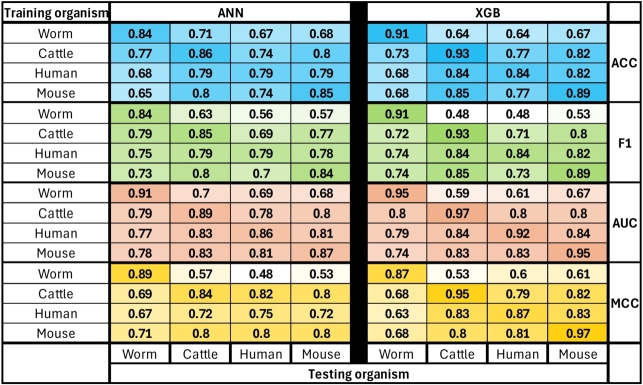
Cross-species-ML classification results. The results are organized into eight heatmaps in a 4X2 matrix. The matrix rows correspond to different metrics, and the columns correspond to different models. Within each heatmap, a cell *(i,j)* represents the mean metric (ACC/F1/AUC/MCC) score of the ten classifiers (ANN/XGB) that were trained on source dataset *i* and tested on target dataset *j*. The diagonal indicates the results of dataset pairs *(i, i)* originating from the same species. The heatmap color scale was adjusted from 0.5 to 1, with 0.5 as the minimum value and 1 as the maximum. ACC = Accuracy.

In the second analysis, denoted as cross-species-ML, we evaluated the performance of cross-species miRNA–target predictions, i.e., the performance of a classifier when applied to interactions from a species different from the one it used for training. We examined all 16 possible combinations, considering each species for training and testing. The results are organized into 8 heatmaps (Fig. 4), as we tested two models (ANN and XGB), with four evaluation metrics. The diagonal in each heatmap represents the intra-species results (as in Fig. 3); other cells represent cross-species results. For example, an XGB model trained on a mouse dataset received an F1-score of 0.74 when tested on a worm dataset. Notably, across most scenarios involving pairs of *human, mouse, and cattle*, the XGB method consistently outperformed the ANN, exhibiting higher values for all evaluation metrics. On the flip side, a distinct pattern emerges for pairs involving *worm* as either the training or testing dataset, where the ANN showed superior performance compared to XGB. Specifically, in 4 out of 6 cases for ACC, AUC, and MCC, and 5 out of 6 cases for F1-score, the ANN outperformed XGB.

As in the previous study [12], combinations that include a worm dataset as training or testing showed reduced performance compared to combinations of other species. Worms have the greatest evolutionary distance from other species in this analysis, which can explain the divergence in miRNA–target interaction rules compared to species that are evolutionary closer, such as human and mouse. Another noticeable phenomenon is the asymmetry of the table. Asymmetry indicates differences in performance for a pair in which one species serves as a training set and another as a testing set compared to the swapped pair. As seen in all heatmaps, a classifier trained on humans performs better when tested on mouse and cattle than in the opposite cases. Due to similar trends between the evaluation metrics and because ACC is more common for binary classification tasks, we used it for the following experiments.

### 3.3 Cross-species-transfer-ML

In the third analysis, denoted as cross-species-transfer-ML, we examined how the ML model can benefit from a small number of interactions from a target species to predict its new interactions. More specifically, we evaluated whether the transfer learning technique improves cross-species classification. The transfer approach involves the fine-tuning of pre-trained models that have been trained on a distinct dataset. In this analysis, the models are first pre-trained on the source species dataset for every combination of <source,target> species pairs. We report two types of analyses: the first examines the effectiveness of transfer learning using ANN, and the second compares different transfer learning methods.

**Figure 5:**
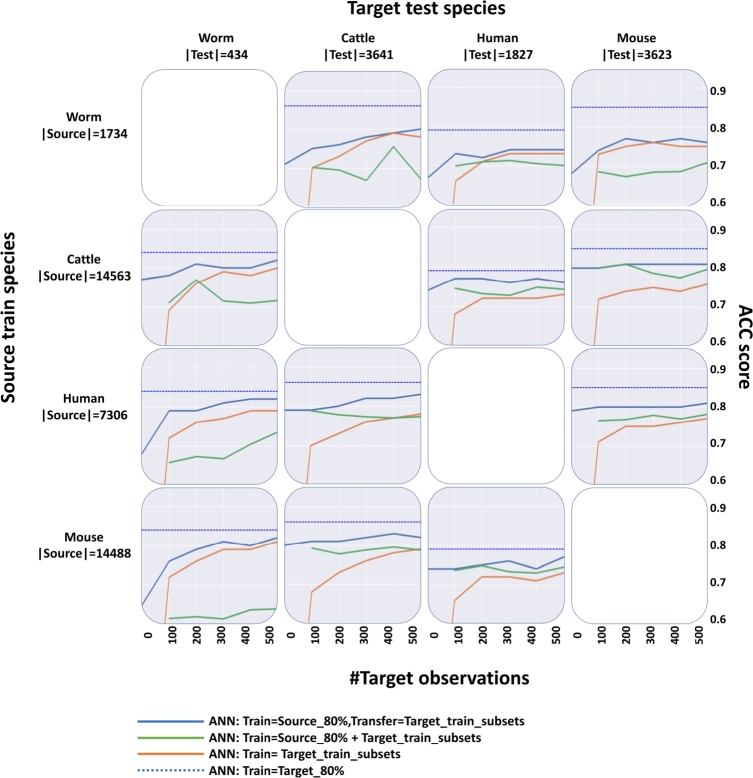
Cross-species-transfer-ML classification results. The results are organized in a 4 by 4 matrix. The matrix rows correspond to the source species, and the columns correspond to the target species. Each cell shows the results for a specific <source, target> species pair, comparing four methods in terms of training data and training approach: **blue line** – ANN transfer method, **green line** – ANN model that uses source observation plus target chunks observation for regular training without transfer, **orange line** – ANN model that uses only chunks of target observations for training, **blue dashed line** – ANN model trained on the complete target species training set (baseline). The right y-axis shows the ACC score, and the x-axis shows the number of target observations used to achieve that ACC score, ranging from 0 to 500. The test set in all cases is composed of a fixed number of observations from the target testing set. ACC = Accuracy.

**Figure 6:**
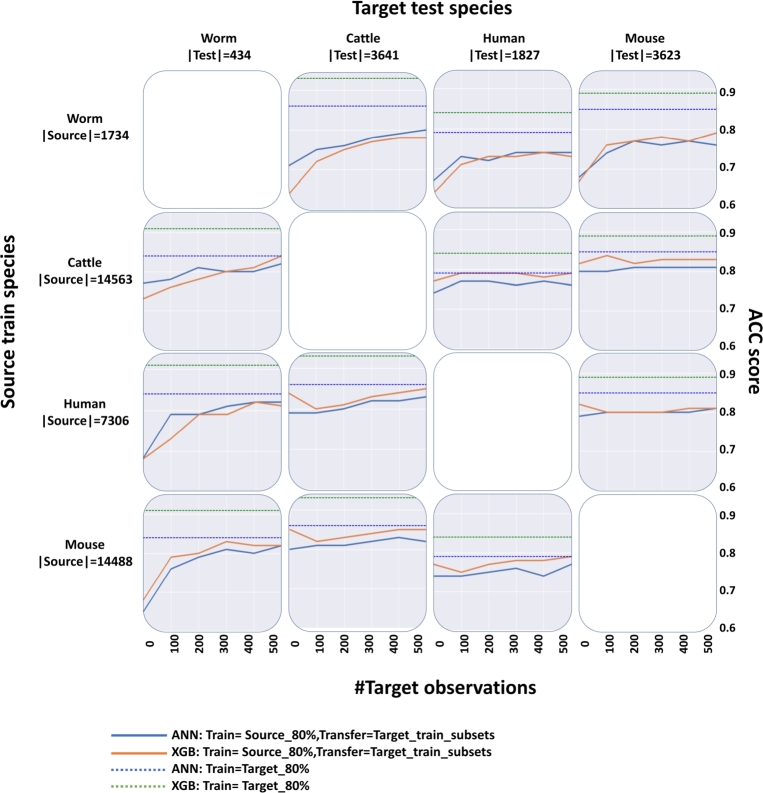
Cross-species-transfer-ML model comparison results. The results are organized as in Fig. 5. Each cell shows the results for a specific <source, target> species pair, comparing four methods: **blue line** – ANN transfer method, **orange line** – XGB transfer method, **blue dashed line** – ANN model trained on the complete target species training set (baseline), **green dashed line** – XGB model trained on the complete target species training set (baseline). The right y-axis shows the ACC score, and the x-axis shows the number of target observations used to achieve that ACC score, ranging from 0 to 500. The test set in all cases is composed of a fixed number of observations from the target testing set. ACC = Accuracy.

To evaluate the effectiveness of the transfer learning approach, we compared four different ANN configurations of the training data and method (Fig. 5): (1) ANN transfer learning method as described in Fig. 2; (2) The same ANN architecture that was trained on both source and target species without the transfer learning technique; (3) The same ANN trained only on a small number of target observations; and (4) As a baseline we trained the ANN using data of target species without transfer learning (equivalent to intra-species-ML). In the transfer learning method comparison, we applied the transfer learning technique over ANN and XGB and compared their performances (Fig. 6): (1) The ANN transfer learning method as described earlier; (2) XGB transfer learning; (3) The ANN baseline when it was trained on data of target species without transfer learning; and (4) the XGB baseline when it was trained on data of target species without transfer learning. The latter two represent results obtained after training on the entire training set of the target species (as in intra-species-ML). In both parts, where relevant, we used the pre-trained models from the cross-species-ML, and the number of observations from the target species used for additional training ranged from 0-500 with steps of 100 observations. For every <source,target> species pair, we used a fixed number of observations from the target testing set for model evaluation.

The results of the first part are shown in Fig. 5. For the <human, worm> pair, the upper limit baseline ACC is 0.84 (blue dashed line). It is achieved for a model that was trained on the complete target species training set and is obtained from intra-ML classification of the worm dataset (Fig. 3). The ANN transfer learning method (blue line) starts with an ACC score of 0.68 when tested on the worm dataset without using any worm interactions during training (the x-axis is 0), representing the cross-ML of <human, worm> pair (Fig. 4). The model performance improves as the number of observations increases, reaching a score of 0.82 when using 500 worm observations. The equivalent model that uses the same observations without the transfer learning (green line) fails to make judicious use of the additional observations and does not improve as we increase the number of observations up to 300. In addition, the model that uses only a small number of target observations for training (orange line) starts with low performance that gradually increases and reaches a maximum score of 0.79 when using the 400 worm observations. In general, the utilization of transfer learning yielded better outcomes when compared to standard ML model training, as we can see by the improved performance of the same ANN architecture. No method reached the upper limit (blue dashed line) in our experiments, but the transfer learning approach came closest, particularly when the target species was a worm.

The comparison of the performance of transfer learning with ANN versus XGB is shown in Fig. 6. While in ANN, we freeze some of the layers, in XGB, there is a follow-up training in which new trees are formed based on the trees of the pre-trained model, known as a partial fit in the XGB algorithm. Looking at the <human, cattle> pair, the XGB transfer learning model (orange line) starts with a 0.84 ACC score, representing the cross-ML result (Fig. 4). After the decrease at the 100-observations point, the performance rises continuously and reaches 0.86 at the final 500 points. In comparison, the ANN transfer method (blue line) starts at 0.79 and always increases to a 0.84 ACC score. The blue dashed line is the ANN upper limit, and the green dashed line is the XGB upper limit, representing the intra-ML performance of ANN and XGB, respectively, when they were trained on the whole target species training set. ANN showed a closer approximation to its upper limit compared to XGB. Furthermore, both transfer models (ANN and XGB) exhibited improved performance as the number of target observations increased, particularly for the worm species. Generally, we can see that XGB outperformed ANN in all cases except for some pairs that include worm as a source or target dataset.

### 3.4 TransferSHAP Feature importance

**Figure 7:**
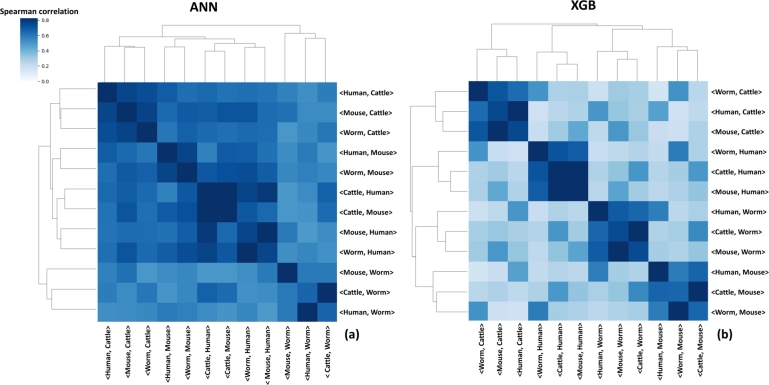
Hierarchical heatmaps of feature importance for transfer learning models. Rows and columns represent transfer learning models of all combinations of <source, target> species pairs, while values represent the Spearman correlation between the transferSHAP values between every two models for (a) ANN and (b) XGB transfer learning models.

Next, we aimed to identify interaction rules most influenced in the transfer learning phase and measure their similarity among different <source, target> species pairs. To that end, we applied our new method TransferSHAP (see Methods) to obtain a list of the most significant features of the XGB and ANN transfer learning models for every <source, target> species pair. We normalized the feature's contribution of every model using MinMax scaling [35] and calculated Spearman correlation between all pairs of normalized vectors and applied hierarchical clustering on the results (Fig. 7).

For both the ANN (Fig. 7A) and XGB (Fig. 7B) models, we identified distinct clusters, where models having the same target species grouped together. This trend was more pronounced for XGB, where rows and columns with the same target species were grouped in triplets. We also observed a similar but less significant pattern in the ANN heatmaps.

## 4 Discussion

Despite the development of novel experimental protocols that have yielded unambiguous interacting miRNA-target datasets, the identification of reliable miRNA targets remains a significant challenge in the field. The technical difficulties associated with these methods have resulted in their limited use for only a few species. Moreover, these methods produce incomplete interaction datasets that cover only a small subset of possible interactions as they are applied to specific cell types or developmental stages. Thus, currently, there is a shortage of comprehensive interaction datasets for many species. As a result, computational approaches have gained increasing interest, particularly for species with limited interaction datasets. In our previous research, we demonstrated that different species share some interaction rules depending on their evolutionary distance [12]. Thus, we hypothesized that species with limited data could benefit from data available for other species.

Transfer learning techniques fine-tune an ML model by transferring the relevant information from the source dataset to the relevant target dataset. As they have been successfully tested and evaluated in various domains, we aimed to evaluate their contribution to transferring miRNA-target rules between species and identify the critical features required to transfer this knowledge.

### 4.1 Intra-merged-species ML

In this study, we chose to work with two advanced ML models, ANN and XGB, and to build our transfer learning methodology on top of them. At first, we merged the eight datasets into four species datasets and evaluated the model's performance prior to applying the transfer technique to miRNA–target prediction. Our results showed that merging the datasets generally improved the performance of both models in most cases except for one exception. Thus merging datasets from the same species can benefit miRNA target prediction. However, the performance may vary depending on the size and nature of the datasets.

### 4.2 Cross-species-ML

We aimed to explore the feasibility of transferring miRNA-mRNA interaction knowledge between species using ML models. Classical ML models such as Random Forest [36], XGB, and Naive Bayes can predict interactions in species other than the one used for training if they are evolutionarily close [12]. We extended this analysis by evaluating the performance of deep learning (ANN) in addition to classical (XGB) models on datasets from four species. Our findings corroborate good performance for species with close evolutionary distances; however, both ANN and XGB struggle to predict interactions between worms and other species. Notably, the ANN model performed better in cases involving significant dissimilarity in interaction rules between species, which occurs in distant evolutionary species. At the same time, XGB outperformed ANN in most cases due to its efficiency in handling small tabular datasets [37].

### 4.3 Cross-species-transfer-ML

To better understand the observed changes in the interaction rules across evolution, we evaluated the effectiveness of transfer learning in fine-tuning the ML models with limited observations in the target species. Initially, we compared the performance of the ANN transfer learning technique with two alternative ANN approaches: the model trained on the target observations only and the model trained on a combination of source and target datasets. Our results revealed that the transfer learning approach yielded superior performance to both alternative approaches. Our findings also showed that the model trained on the target observations only required approximately 500 observations to achieve comparable performance to the ANN model with transfer learning. Interestingly, adding more target observation chunks to the models trained on source and target without transfer learning did not improve or even degrade the results in some cases, especially those involving worm and cattle species. Furthermore, we observed similar trends for XGB transfer learning performance. However, the ANN model outperformed XGB in transferring data from and to worm species, while XGB was more efficient in learning and predicting interactions in all other cases. Additionally, we observed that the ANN transfer learning managed to get closer to its upper limit than XGB, meaning that the transfer learning using the ANN model achieved almost the same accuracy as the ANN model trained on the entire target training set. This finding suggests that the ANN model can adapt to the new dataset more easily than XGB.

### 4.4 Essential interaction rules

The proposed methodology in this study, TransferSHAP, aimed to identify the vital features of transfer learning. Interestingly, when we applied this method to all <source, target> species pairs and clustered the outcomes based on the Spearman correlation, pairs of the same target were clustered together independent of the source species used to train the models. This indicates that the target species has a dominant influence on the feature importance patterns for transfer learning. We also observed that the clusters were more clearly defined in the XGB results compared to the ANN results. This may be due to the boosting algorithm used by XGB, which selects the least features to maximize accuracy.

### 4.5 Limitations and future work

The present study incorporated fine-tuning into ANN and XGB models, utilizing hyperparameters optimized for source species models and using them for transfer learning models. However, it is possible that this set of hyperparameters is not optimal for the latter models, particularly when the transfer phase uses a small number of observations from the target species. Future work could examine the impact of hyperparameter fine-tuning at the transfer learning training on the model's performance.

The explainability tool, TransferSHAP, introduced in this study isolates the contribution of transfer learning alone, excluding the influence of source models. It is developed for estimating feature importance in tabular dataset binary tasks. In its current version, TransferSHAP calculates the absolute value of feature importance, irrespective of the predicted class. A potential area for future work lies in studying the differences in feature importance between positive and negative classes, offering more insights into interaction rules across evolution. Additionally, there is an opportunity to extend the tool's capabilities to support multiclass classification models.

## 5 Conclusions

Our study presents novel contributions to the field of miRNA–mRNA interaction prediction. We proposed a methodology to enrich the MTI prediction, especially for species with limited data, and improved their results significantly. Additionally, we introduced the TransferSHAP method for measuring the feature importance of transfer learning with tabular datasets. Using that method, we identified what features were essential and similar in transferring knowledge between species. Our findings highlight the effectiveness of transfer learning in improving the performance of close and distant evolutionary species. Interestingly, we observed that the ANN model outperformed the XGB model in cross-species prediction, with and without transfer learning, particularly in the case of worm interactions. This suggests that transferring knowledge between distant species requires more complex ML methods. Based on our research and the specific datasets used for the MTI task, we found that the effectiveness of the transfer learning method is limited to a certain number, which in our case was determined to be 500 observations. The transfer learning method was known to be effective in many domains, and in this study, we discovered that it could also be used for the MTI task.

## Funding

This work was supported by the 10.13039/501100003977Israel Science Foundation [520/20]. The funding agency had no role in study design, data collection, analysis and interpretation, or manuscript preparation.

## CRediT authorship contribution statement

**Eyal Hadad:** Writing – review & editing, Writing – original draft, Methodology, Investigation, Formal analysis, Data curation, Conceptualization. **Lior Rokach:** Writing – review & editing, Supervision, Methodology. **Isana Veksler-Lublinsky:** Writing – review & editing, Writing – original draft, Supervision, Methodology, Funding acquisition, Conceptualization.

## Declaration of Competing Interest

The authors declare that they have no known competing financial interests or personal relationships that could have appeared to influence the work reported in this paper.

## Data Availability

The datasets supporting the conclusions of this article are available in the Zenodo repository: https://zenodo.org/doi/10.5281/zenodo.10435941. The code is available in the following repository for the transfer learning experiments: https://github.com/IsanaVekslerLublinsky/TransferLearningMTI.git. Additionally, the code for TransferSHAP can be found at https://github.com/IsanaVekslerLublinsky/TransferSHAP.git.
